# Autonomous Drilling and the Idea of Next-Generation Deep Mineral Exploration

**DOI:** 10.3390/s25133953

**Published:** 2025-06-25

**Authors:** George Nikolakopoulos, Anton Koval, Matteo Fumagalli, Martyna Konieczna-Fuławka, Laura Santas Moreu, Victor Vigara-Puche, Kashish Verma, Bob de Waard, René Deutsch

**Affiliations:** 1 Department of Computer Science, Electrical and Space Engineering, Signals and Systems, Luleå University of Technology, 971 87 Luleå, Sweden; anton.koval@ltu.se (A.K.); mafum@dtu.dk (M.F.); 2 Department of Electrical and Photonics Engineering Automation and Control, Technical University of Denmark, Elektrovej, 2800 Kongens Lyngby, Denmark; lsamo@dtu.dk (L.S.M.); vvipu@dtu.dk (V.V.-P.); s230015@student.dtu.dk (K.V.); 3 Faculty of Geoengineering, Mining and Geology, Wrocław University of Science and Technology, 15 Na Grobli Street, 50-421 Wrocław, Poland; martyna.konieczna-fulawka@pwr.edu.pl; 4 Spectral Industries B.V., Schieweg 15A, 2627 AN Delft, The Netherlands; waard@spectral-i.com; 5 Epiroc Rock Drills AB, Klerkgatan 21, 701 91 Örebro, Sweden; rene.deutsch@epiroc.com

**Keywords:** autonomous drilling, SLAM navigation, modular robotic systems, underground mining automation, LIBS analysis, LoRaWAN networks, exploration drilling, robot collaboration, real-time rock sensing

## Abstract

Remote drilling technologies play a crucial role in automating both underground and open-pit hard rock mining operations. These technologies enhance efficiency and, most importantly, improve safety in the mining sector. Autonomous drilling rigs can navigate to pre-determined positions and utilize the appropriate parameters to drill boreholes effectively. This article explores various aspects of automation, including the integration of advanced data collection methods that monitor the drilling parameters and facilitate the creation of 3D models of rock hardness. The shift toward machine automation involves transitioning from human-operated machines to systems powered by artificial intelligence, which are capable of making real-time decisions. Navigating underground environments presents unique challenges, as traditional RF-based localization systems often fail in these settings. New solutions, such as constant localization and mapping techniques like SLAM (simultaneous localization and mapping), provide innovative methods for navigating mines, particularly in uncharted territories. The development of robotic exploration rigs equipped with modules that can operate autonomously in hazardous areas has the potential to revolutionize mineral exploration in underground mines. This article also discusses solutions aimed at validating and improving existing methods by optimizing drilling strategies to ensure accuracy, enhance efficiency, and ensure safety. These topics are explored in the context of the Horizon Europe-funded PERSEPHONE project, which seeks to deliver fully autonomous, sensor-integrated robotic systems for deep mineral exploration in challenging underground environments.

## 1. Introduction

Both underground and open-pit mines are undergoing a transformation due to advancements in automation and diagnostic systems. This change is particularly significant in the underground mining sector, as it reduces the need for human workers to operate in hazardous environments. This paper discusses the implementation of advanced autonomous solutions in remote drilling technologies and the operation of drill rigs. The current drilling systems can navigate to specific drilling locations, drill at defined angles, and adapt to various geological conditions by autonomously changing drill rods or bits as needed [1,2,3]. By collecting real-time data on key drilling metrics—such as penetration rates and hydraulic pressures—it is possible to create complex 3D models of subsurface rock properties. These parameters are essential for planning the most effective extraction processes [4]. The increased development of automation is driven by the widespread implementation of AI (artificial intelligence) algorithms in mining, particularly in the operation of mining machines. This conversion process incorporates aspects such as robotic processes, algorithm automation, and AI integration [5]. However, this transformation presents challenges, especially related to traditional navigation methods. Navigation methods that rely on RF (radio frequency) signals face limitations due to signal propagation issues in underground environments. The absence of ground control points makes it nearly impossible to use conventional correction methods. To address this, robotic platforms often employ a combination of IMUs (inertial measurement units) and magnetic induction systems, allowing for effective navigation even in areas that are not fully mapped [4]. Additionally, the integration of visual and LIDAR sensors, similar to the simultaneous localization and mapping (SLAM) algorithm, enables the creation of three-dimensional models of the surroundings, enhancing overall operational safety [6]. However, visual SLAM is less reliable in low-light conditions, where onboard illumination may be inadequate or may cause reflections that hinder feature tracking. While LIDAR-based SLAM is generally resilient to lighting issues, it can be affected by dust, fog, and sparse geometric features, negatively impacting scan matching accuracy and loop closure detection [7]. Recent studies indicate that LIDAR SLAM can accumulate mapping errors exceeding 1 m per 100 m in a homogeneous linear environment, whereas radar-based SLAM maintains an accuracy of less than 0.5 m under the same conditions [8]. In dusty environments, experiments conducted over a 7 km trajectory in a hard-rock mine reported a median mean Euclidean error of 122.4 m when using laser-only localization, while fusing LIDAR with a camera reduced the error to just 1.32 m [9].

This article presents the conceptual framework, technological background, and implementation strategies developed within the PERSEPHONE project, which aims to revolutionize deep mineral exploration through the deployment of autonomous, sensor-integrated robotic systems. The focus is on integrating drilling automation, real-time rock characterization, advanced SLAM-based navigation in GPS-denied environments, and modular robotic platforms capable of operating in confined and hazardous underground settings. By combining onboard sensing, intelligent data processing, and multi-agent coordination via mesh communication networks, PERSEPHONE seeks to enhance both the efficiency and the safety of mineral exploration operations. The article outlines the technical architecture, component technologies, and mission planning strategies that support fully autonomous drill planning and execution, contributing to the next generation of intelligent and sustainable mining solutions.

## 2. Drilling Systems and Peripherals

Remote drilling technology is leading the way in automation within the hard rock mining industry. In many mines, drill rigs now operate autonomously, moving to new drilling positions and using auto-navigation to reach the designated collar position for new holes. These machines are capable of drilling at specified angles and orientations.

Before planning drilling operations, it is essential to understand the drill rig’s workspace to ensure that it can operate autonomously [7,10]. Additionally, these machines can automatically add drill rods and change drill bits when necessary. Recent advancements have enabled the collection of mechanical data during the drilling process, including information on drilling speed, penetration rate, rotation speed, as well as hydraulic and physical pressure [11,12].

Typically, this data is automatically attributed, helping to create 3D models of rock hardness, which can then be translated into geological models. The conversion of hydraulic pressure and penetration rate into rock hardness values is based on empirical regression models calibrated with known rock samples. These models are refined using supervised machine learning techniques, such as random forest and support vector regression (SVR), trained on datasets that combine penetration rate, torque, and bit wear. Limitations include sensor drift, variability in drill bits, and reduced accuracy in fractured zones.

The journey of business process transformation, from basic automation to artificial intelligence, is clearly defined and varies across a wide range of industries. The stages of this transformation are as follows:

- Basic automation: In this stage, all parts of the processes are triggered by humans, governed by very simple rules, with no integration with other systems.
- Robotic process automation (RPA): This involves more than one system, where parts of the process may still be triggered by humans, while mundane tasks are handled by interoperable processes. Although the rules remain simple, this allows for higher volumes of work to be processed, though it is still limited to structured data and operates at an enterprise level.
- Enhanced process automation: Here, human input is augmented by basic analytics and decision support. Unstructured data comes into play through intelligent document processing and optical character recognition, with integration extending to basic web applications, such as company search engines.
- Algorithmic automation: This stage enables complex data-based decision-making, where humans select the best option informed by predictive and prescriptive analytics. It may involve rudimentary machine learning that suggests optimal decisions while incorporating unstructured and big data, thereby increasing the complexity of algorithms. Advanced integration with the internet of things (IoT) is also possible.
- Artificial intelligence: At this level, cognitive technology mimics human decision-making capabilities, offering end-to-end autonomy that includes reasoning and hypothesizing. This stage involves deep learning linked to neural networks, as well as speech recognition and generation, augmented reality, and virtual reality.

These stages of transformation are well articulated and defined, reflecting variable levels of transformation across a wide spectrum of industries [13,14,15].

In the PERSEPHONE project, the current drilling systems operate at the algorithmic automation level. These systems incorporate advanced sensor suites, such as LIBS, IMUs, and LIDAR, along with predictive analytics for drilling performance. They also feature partial autonomous decision-making capabilities based on real-time data. While the development of cognitive AI is underway—particularly in adaptive drilling strategies and autonomous navigation in unknown environments—the project has not yet achieved full “Artificial Intelligence” autonomy as defined by the five-level framework.

Lab testing and modular integration trials have shown promising robustness under controlled conditions. However, real-world field trials in operational mining environments are planned for the next phase of development. These trials aim to evaluate the systems’ resilience, fault tolerance, and mission autonomy in real-world scenarios.

## 3. Navigation of Autonomous Machines in Uncharted Areas

Navigating underground environments presents numerous challenges. One significant issue is that technologies like GPS struggle to function effectively due to the difficulty of radio frequency (RF) signal propagation at such high frequencies [16]. Additionally, it is often impractical to install ground control points (GCPs) within a mine, which limits the application of correction methods using optical techniques.

To accurately localize the robot, a system that does not rely solely on RF communication is essential. One recommended approach is to employ the classic dead reckoning method in combination with an inertial measurement unit (IMU) and magnetic induction (MI). Magnetic induction involves measuring the magnetic field using a magnetometer, which helps create a map based on known magnetic field intensity values when combined with readings from an accelerometer. External limit-switching pads can define the mine’s tunnel boundaries and safeguard the robot from potential collisions. The accuracy of localization heavily hinges on variations in the magnetic field, which should be sufficiently pronounced, given the abstract geometry of the mine tunnels. However, the MI method is only effective in pre-mapped environments and can be influenced by paramagnetic materials present in ores [17].

Another viable strategy is to utilize a camera or LIDAR sensor alongside an accelerometer or the “structure from motion with optical flow” technique to create a 3D representation of the mine through photogrammetry or direct mapping [18]. By employing simultaneous localization and mapping (SLAM) algorithms [19], a camera can autonomously navigate a mine. Combining these two approaches allows for the creation of a 3D map with known magnetometer values, enabling the robot to use SLAM for unexplored areas while relying on magnetometer readings in previously mapped regions.

While both methods operate without relying on RF communication, effective and safe navigation in underground environments still necessitates a communication system. In scenarios where robots depend solely on MI in mapped areas, they cannot perceive each other’s presence, which potentially leads to erratic behavior during collisions. Robots equipped with vision-based sensors, such as cameras, can be marked with unique IDs to facilitate the implementation of collision prevention algorithms. However, this solution falls short if a robot cannot visually identify the ID of an approaching robot. Radio- frequency identification (RFID) systems provide a practical solution for detecting tagged personnel or assets. However, their performance can be compromised by signal interference from nearby metallic structures, which are common in underground machinery. This interference can negatively impact the distance estimation accuracy and reduce the overall reliability of the system [20].

On the other hand, radar-based perception presents a promising alternative, as it operates effectively in challenging environments where LiDAR and vision systems may fail. Radar can reliably detect both static and dynamic obstacles in underground tunnels [21].

In addition to assisting with collision avoidance, it is crucial to implement communication capabilities that allow robots to share navigation information with each other when they cross paths. This shared data, which can include 3D maps and point cloud information, can be transmitted via RF communication. In any situation, a “dead reckoning” approach is inevitable for navigation in non-mapped areas unless a high-FPS camera and a computable algorithm can generate the map [22]. It is unrealistic to expect robots to have the processing power required to manage multiple point clouds and 3D meshes efficiently. Therefore, a server must handle this processing on their behalf. To achieve this, point clouds need to be transmitted back to the server, and the server must send commands to the robots [23]. This process requires the use of repeaters in large mines. By employing signal repeaters, it is possible to cover extensive networks of tunnels [24].

The research mentioned earlier focuses on the use of WLAN networks. However, WLAN operates at a high frequency, which limits its ability to penetrate soil. This necessitates positioning repeaters in line-of-sight locations. Additionally, the range of a WLAN repeater is typically insufficient, resulting in a great number of repeaters and increased overhead in communication packets. In contrast, using LoRa WAN instead of WLAN is more effective in underground environments, as LoRa WAN operates at a lower frequency enabling it to penetrate a reasonable amount of soil [25]. LoRaWAN can provide data rates ranging from 3 to 50 kbps, depending on the channel bandwidth and spreading factor. In urban areas, as well as rural settings, these networks can cover distances of 5 to 20 km, which is particularly advantageous for subterranean environments. Additionally, there are various methods for implementing multi-hop transmission with LoRaWAN, allowing for the creation of an ad hoc network, as discussed in this paper [26]. However, much of the existing research on ad hoc networks has focused on a limited number of nodes and low traffic volumes. This suggests a need for further investigation into deploying a LoRaWAN mesh network with a larger number of nodes.

Based on the information provided, it can be concluded that a combination of a magnetometer (when feasible), a camera, and an inertial measurement unit (IMU) will accurately enable navigation for a robotic rig in a mine. The optical system could serve as the primary navigation tool, while the magnetic and IMU sensors would help minimize errors in previously mapped areas. Additionally, it is essential for other robots to deploy LoRa WAN repeaters when the signal strength decreases, ensuring that communication with the server remains accessible.

Localizing mobile legged robots, such as robotic dogs, in underground tunnels presents unique challenges due to GPS-denied environments, limited visual cues, and unstable radio frequency (RF) conditions. To achieve accurate localization, it is crucial to rely on tightly coupled sensor fusion, which includes IMU, LiDAR, and magnetic simultaneous localization and mapping (SLAM) with local mapping nodes. Stable communication, necessary for synchronization and safety, is supported by LoRaWAN mesh networks. However, high-throughput tasks, such as image transfer, require the use of buffer nodes or edge-computing repeaters.

Therefore, to enable robust and energy-efficient wireless communication in underground environments, scientists are working on adopting in the PERSEPHONE system a LoRaWAN-based architecture. The primary communication channel operates in the 868 MHz ISM band (EU868 standard), with optional fallback to the 433 MHz range in high-attenuation conditions. LoRaWAN is selected for its superior propagation characteristics in non-line-of-sight conditions and low energy footprint. Depending on the spreading factor and channel conditions, data rates range from 0.3 to 50 kbps. Receiver sensitivity extends to approximately −137 dBm, while the system design defines an operational signal strength threshold of −90 dBm, below which message retransmission or automatic repeater deployment is triggered. To extend coverage and ensure mission continuity in long or branched tunnels, repeaters will be deployed to avoid quality drops.

## 4. Next-Generation Deep Mineral Exploration Drilling Systems and Autonomous Drill Planning and Execution from Onboard Sensing of Rock Face Characteristics

### 4.1. Modular System for the Deployment of Exploration Drilling Rigs and Measurement Systems, Leveraging Existing Robotic Solutions

**Figure 1:**
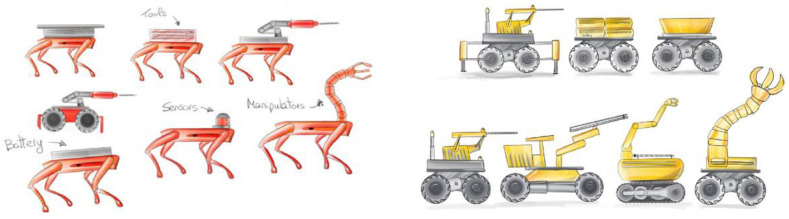
Various concepts of operating scenarios of an autonomous modular machine for exploration and extraction of deposits.

The Next-Generation Deep Mineral Exploration Drilling Systems project focuses on developing technologies that enable autonomous machines to operate in areas of a mine that are unsafe or inaccessible for human workers. The project involves modular systems consisting of robotic mobile bases, manipulators, end effectors, and sensors (Figure 1). These systems will allow users to safely and efficiently assess conditions in unexplored regions.

To evaluate the performance of these systems, several key metrics can be defined, as follows:

- Traversal success rate: The percentage of tunnel sections completed without human intervention.
- Exploration coverage rate: The area mapped per unit of time (e.g., square meters per minute).
- Energy consumption: The energy required to complete specific tasks.
- Reconfiguration time: The average time required to switch between modules or tools.

These metrics can also be used to directly compare the performance of autonomous modular systems against that of traditional manual operations, helping to quantify how these technologies enhance the efficiency, safety, and scalability of various mining activities.

Additionally, multiple robots will be linked or combined to provide various functionalities for exploration drilling missions. For instance, they could supply additional power to the drilling tools or replace drill extensions. The early-stage development of this technology will involve

- Research into existing robotic drilling technologies and modular systems.
- CAD design.
- Prototyping and testing conducted in a laboratory environment.
- Integration of hardware and software for the prototyped components into existing robotic solutions.

**Figure 2:**
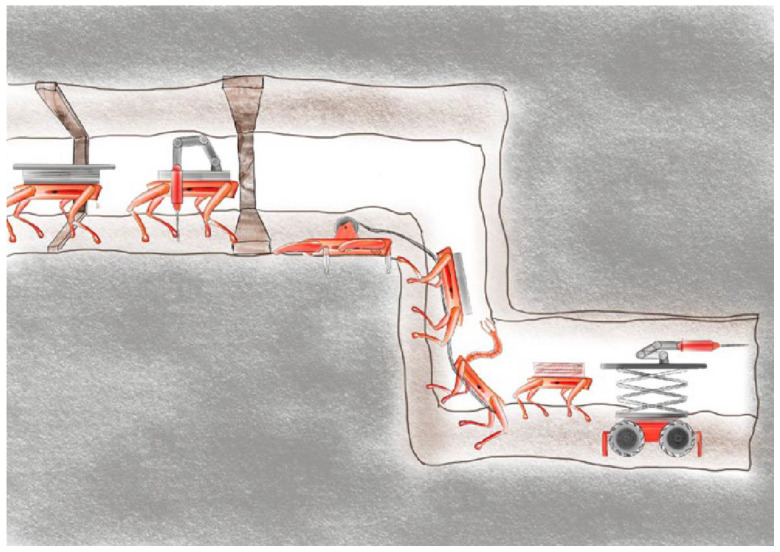
Conceptual mining machine reaching deposit unreachable by standard mining operations.

The development of a new modular system will significantly enhance the current state of the art (SoTA). First, delivering drilling rigs to hard-to-reach and hazardous areas will enable continuous exploration even in harsh environments (see Figure 2).

**Figure 3:**
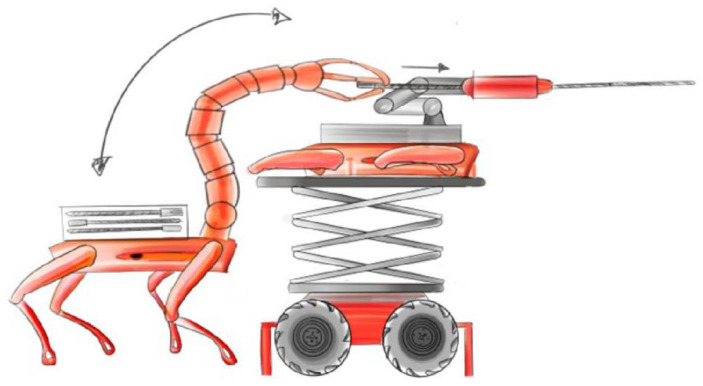
Concept of modular prospection and excavation machine supported by another autonomous robotic machine.

Secondly, the implementation of new advances in modular robotic systems (Figure 3) will enable versatile and efficient exploration and mining operations in challenging environments.

Finally, a fleet of modular ground robots carrying sensors and mechanical elements are necessary to guarantee

(1) Traversability of the mine.
(2) Distribution of loads and transportation of electrical and drilling mediums.
(3) Physical collaboration for precise exploration drilling.

Furthermore, multiple robots will be linked or combined to provide one or more functionalities for exploration drilling missions. In addition to enhancing operational safety and efficiency, the modular design also improves maintainability and adaptability in complex mining environments. From a maintenance perspective, faulty or worn-out modules—such as locomotion units, sensors, or tools—can be individually replaced or repaired without requiring the entire system to be dismantled. This reduces the downtime and supports long-term deployment in harsh underground conditions.

Regarding adaptability, the modular framework allows for the creation of different functional agents using a shared robotic base. By swapping sensor packages, end effectors, or support tools, the same platform can be reconfigured to perform a variety of tasks, such as inspection, drilling preparation, or load transport, depending on the specific mine geometry or operational needs. This flexibility makes the system scalable and applicable across a wide range of underground exploration scenarios.

Deploying such a robotic fleet for autonomous drilling also necessitates robust control software to coordinate complex multi-agent behaviors. Each robot may fulfill a distinct role, such as drilling, supplying power, or conducting environmental scans, which requires high-level planning, task allocation, and dynamic reconfiguration in response to environmental changes or hardware faults. Recent examples, such as the CERBERUS and CoSTAR systems developed for the DARPA Subterranean Challenge, highlight modular and scalable software architectures that enable heterogeneous robots to collaborate autonomously in GPS-denied and cluttered environments. For instance, CoSTAR’s NeBula integrates belief-aware planning and distributed task execution [27], while CERBERUS employs an adaptive autonomy architecture, known as resilient autonomy, allowing both legged and aerial platforms to dynamically switch roles (e.g., exploration, mapping, communication relay) according to environmental constraints, sensor reliability, and mission demands [28].

### 4.2. Autonomous Drill Planning and Execution Systems That Utilize the Onboard Sensing of Rock Face Characteristics

To fully automate the exploration and extraction of mineral deposits, it is essential to equip machines with devices that enable the collection of real-time data on the composition of the rock face. Information about the geometry of the rock face and the arrangement of geological layers will serve as the basis for the ongoing planning of the drilling operations and for adjusting drilling and blasting patterns.

One of the key objectives of the PERSEPHONE project is to develop autonomous systems for drill planning and execution that utilize onboard sensors to assess rock face characteristics. This technology will integrate sensors into drilling equipment to collect real-time data on rock properties and the surrounding drilling environment.

To achieve these goals, the project team will perform the following tasks:

- Conduct theoretical research to understand the principles of onboard sensing and its application in autonomous drilling.
- Investigate and adapt existing sensor technologies and data processing algorithms for the specific use cases outlined in the PERSEPHONE project.
- Establish seamless communication and coordination between robotic systems and tools, thereby enhancing their ability to perform complex maneuvers with precision and efficiency.

These measures will enable us to autonomously plan and execute drilling operations by analyzing real-time sensor data from the drilling equipment. The innovative aspect of the proposed solution is its ability to adjust parameters based on rock characteristics to optimize drilling efficiency and effectiveness.

The PERSEPHONE project aims to achieve high accuracy in the system for autonomous drill planning and execution, using the onboard sensing of rock face characteristics. It is also expected that, under simulated conditions, the system will respond accurately to various geological scenarios and drilling conditions.

The development of this technology will significantly advance the current state of the art by allowing autonomous drilling systems to gather and utilize real-time data on rock characteristics and working geometry for data-driven decision-making. This will undoubtedly enhance drilling efficiency, reduce dependence on manual intervention, and improve safety by providing valuable insights into geological conditions during drilling.

### 4.3. PERSEPHONE Autonomous Drill Planning and Execution Approach

This section outlines the architecture and mission concept for a fully autonomous robotic fleet designed to perform exploration drilling in challenging underground environments. This approach complements the previously introduced modular and sensor-integrated systems.

To design the fleet’s operation effectively, it is crucial to understand the scenario constraints. Since the mission targets deep, abandoned mines, the following constraints influence the robotic architecture:

A dynamic and partially unknown environment: The local mine geometry and layout are assumed to be unknown before the mission execution. The system must rely solely on online mapping and localization using onboard sensors.

No external infrastructure: The lack of communication networks or localization beacons requires the use of self-sufficient onboard systems and a decentralized ad hoc wireless mesh network for inter-robot communication.

Confined operational space: The typical mine tunnel dimensions are limited to 2 m × 2 m, necessitating compact, highly maneuverable robots along with specialized manipulation strategies.

Harsh conditions: The system must operate reliably in environments with poor visibility, airborne dust, uneven terrain, moisture, and potentially toxic gases.

These constraints inform the design and modularity of the proposed robotic fleet, ensuring robustness, adaptability, and full autonomy.

The PERSEPHONE autonomous drilling concept employs a heterogeneous multi-agent robotic fleet, with each agent assigned a specialized role. All platforms are modular, equipped with onboard sensing systems, and capable of performing their tasks independently:

- Deployer robot: A ground vehicle equipped with a six-DoF industrial manipulator and an array of sensors (e.g., LiDAR, cameras, IMU, LIBS). Its roles include 3D environment scanning, deployment site analysis, and manipulation tasks such as placing and servicing the drilling tool.
- Supplier robot: A logistics agent with a high payload capacity, designed to transport essential resources like power units, drilling consumables, and water, or even the stinger robot. It acts as a mobile depot that dynamically supports the drilling operation.
- Stinger robot: A compact, custom-built drilling agent with a deployable anchoring system and an integrated drilling unit. It is capable of self-anchoring, performing multi-hole drilling operations, and adjusting the drilling parameters based on local sensor feedback.

**Figure 4:**
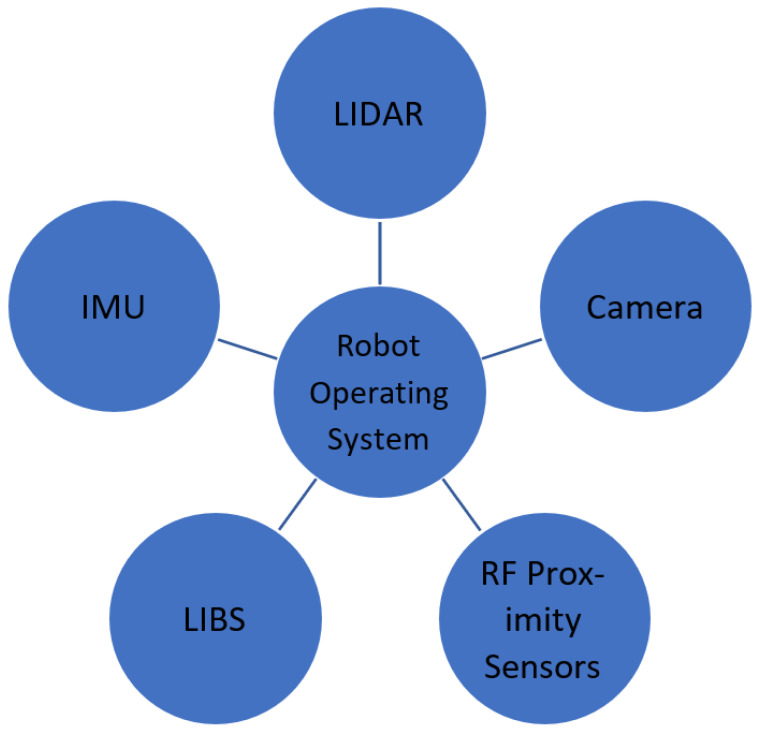
Sensor integration architecture for autonomous navigation and analytics.

Figure 4 demonstrates how different sensor modalities are synchronized through a unified software stack, enabling integrated SLAM and real-time decision-making. Sensor fusion enhances operational redundancy and improves perception in complex underground environments.

Each robot is designed with modularity in mind, allowing for flexible reconfiguration and collaboration in space-constrained, high-risk environments.

The drilling operation is executed through a structured and adaptive sequence of collaborative actions. Each mission begins with a coarse estimate of the drilling location and unfolds in the following phases:

- Environment mapping: The deployer robot scans the local geometry to create a 3D map of the area.
- Deployment site selection: Using its onboard perception, the deployer analyzes the terrain to identify suitable locations for anchoring the stinger robot.
- Stinger robot deployment: The deployer retrieves the stinger robot (either from itself or from the supplier), navigates to the chosen site, and performs precise pick-and-place operations for deployment. Once the stinger robot is successfully placed, the deployer communicates the completion to it, enabling further actions.
- Anchoring and resource supply: After receiving clearance from the deployer, the stinger robot begins deploying its anchoring system and confirms stability using its onboard sensors. The deployer or supplier supplies power, water, and drilling tools as needed, based on requests from the stinger robot.
- Drilling execution: The stinger robot conducts adaptive drilling, utilizing local sensing to adjust to material properties or disturbances. It can reposition its drilling head to create multiple holes per deployment.
- Repositioning (if required): If additional drilling is needed beyond the robot’s reach or if there is a failure in any other phase of the mission, the stinger robot communicates the issue to the deployer. The deployer will then retrieve and relocate the stinger robot to a new site, repeating the deployment and drilling cycle.
- Mission completion: After the drilling plan is completed, the fleet consolidates, stores the stinger robot, and awaits further tasks or extraction.

**Figure 5:**
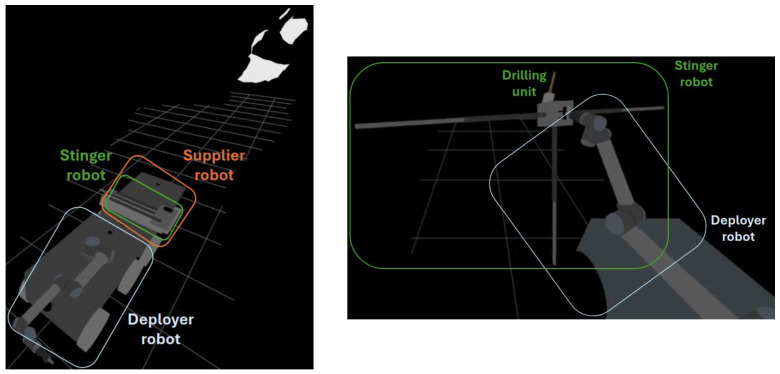
Robotic fleet concept for autonomous drilling (**left**) and stinger robot deployment and anchoring (**right**).

Throughout the mission, the coordination between sequential actions occurs via communication over the local mesh network, ensuring synchronization and shared situational awareness across robots. In the case of any failure in any of the mission phases, the repositioning phase initiates through communication between deployer, stinger, and supplier. Moreover, the mission phases are sequential, and each mission phase corresponds to the actions of a single agent. The transitions between mission phases are explicitly validated through inter-agent communication, guaranteeing safe and efficient collaboration without centralized control. Figure 5 below presents concept of robotic fleet for autonomous drilling and stinger robot deployment and anchoring.

Each failure scenario follows a consistent failure response → adaptation → restart or fallback → notification pattern, ensuring distributed fault tolerance without central control, as presented below in the Table 1.

## 5. Real-Time Rock Characterization and Detection of Rock Mechanical and Geophysical Properties Supported by Advanced Online Analytics Systems for the Planning and Characterization of Near-Mine Exploratory Drilling

**Figure 6:**
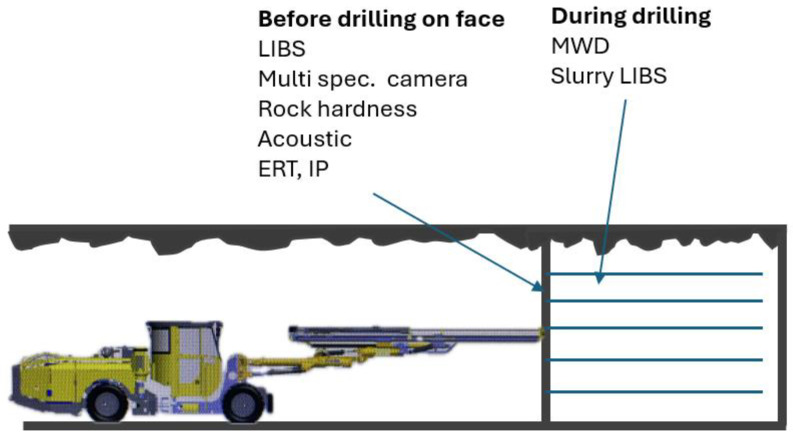
Parameters collected before and during borehole drilling.

Real-time rock characterization, supported by online analytics systems, is the most extensive module of the PERSEPHONE project due to the complexity of the technologies involved [29,30]. The approach developed as part of the project is based on the assumption that a measurement system equipped with a series of sensors will continuously collect and transmit information during both the preparation for drilling and the drilling process itself. On the Figure 6 below, two LIBS scenarios are described, and one will be selected for in-field testing as part of the PERSEPHONE project.

### 5.1. LIBS Slurry Analyzer

LIBS (Laser-induced breakdown spectroscopy) is an analytical technique that employs a high-power laser beam to determine the chemical composition of a target material. This technique has been in use for quite some time and has proven effective in various academic and industrial fields, including mining, recycling, and manufacturing [31].

**Figure 7:**
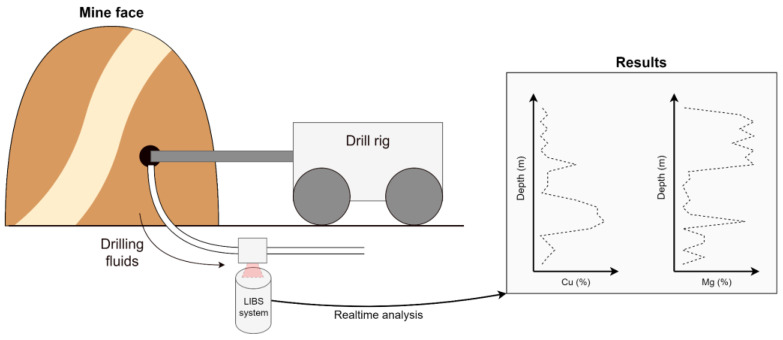
Scheme of LIBS slurry analyzer.

The concept of the LIBS drilling slurry analyzer is illustrated in the figure below (Figure 7). During the drilling process, drilling fluids are collected from the drill hole. These collected fluids are analyzed in real time by a LIBS system to ascertain their chemical composition. The data obtained from this analysis can be utilized to create a depth profile of the composition of the slurry located behind the mine face.

The selection of laser-induced breakdown spectroscopy (LIBS) as the primary method for slurry analysis in the PERSEPHONE project is based on its suitability for real-time, in situ applications in harsh underground environments. Unlike methods such as X-ray fluorescence (XRF) or mass spectrometry, LIBS allows for direct elemental analyses without the need for extensive sample preparation, shielding, or a controlled laboratory setting. An early-stage LIBS prototype for slurry diagnostics was developed and tested under laboratory conditions within the H2020 ROBOMINERS project, demonstrating its technical feasibility and laying the groundwork for further development. Although no formal benchmarking was conducted at that time, the operational simplicity, compact design, and rapid acquisition time of LIBS make it a strong candidate for integration into mobile robotic platforms [32,33]. This prototype can serve as a starting point for additional development in PERSEPHONE. Laboratory studies can help improve our understanding of detection limits in slurries, measurement parameters, temporal resolution, and other technical enhancements to make the system more viable in mining environments.

LIBS has been actively researched in academia for analyzing fluids, but this is typically done in highly controlled laboratory conditions with careful sample preparation. Adapting this technology for harsh mining environments is a significant challenge, but if successful, could revolutionize the drilling technology by providing early data on the composition of the rock behind a mine face before blasting.

To justify the selection of laser-induced breakdown spectroscopy (LIBS) for integration with autonomous drilling platforms in the PERSEPHONE project, a comparison was conducted with other widely used elemental analysis techniques—namely, X-ray fluorescence (XRF) and mass spectrometry (e.g., ICP-MS). While each method offers distinct advantages, their applicability varies significantly depending on analytical goals, environmental constraints, and operational context. The following Table 2 summarizes key performance characteristics and operational considerations relevant to underground mining environments.

Still, it has to be pointed out that, as highlighted above, the current state of technology is still immature and relies primarily on laboratory experiments. However, early and promising results have been achieved with brines and dense slurries using an experimental prototype. By the end of the project, we aim to have a new, validated setup ready and potentially conduct initial field experiments for various applications.

### 5.2. LIBS Mine-Face Scanning

**Figure 8:**
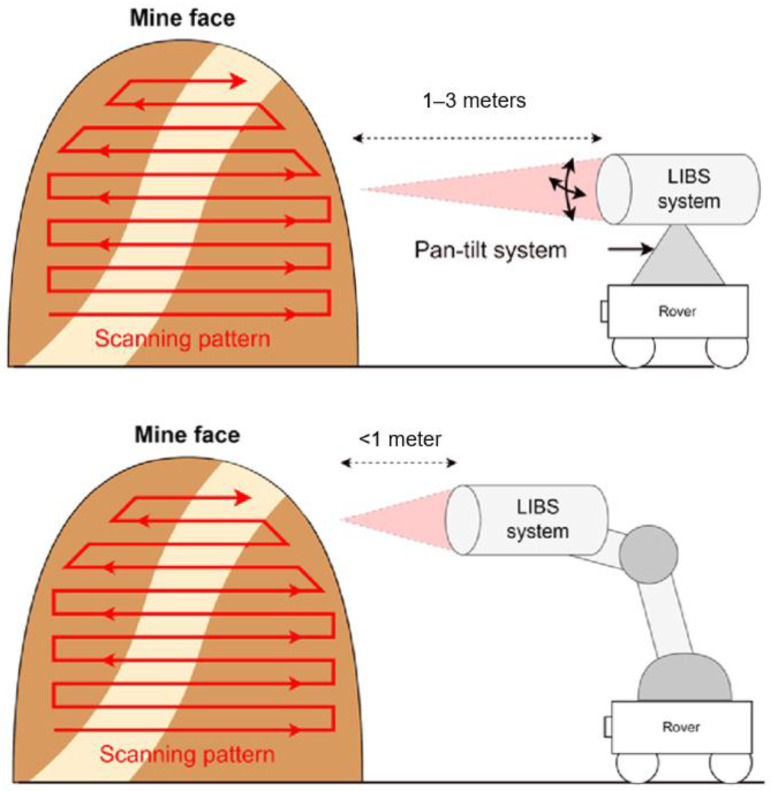
LIBS mine-face scanning (the red arrows show the direction of the scan).

A LIBS (laser-induced breakdown spectroscopy) sensor could be developed to scan a mine face from a distance, creating a detailed map of the area. This map can help visualize the ore body and identify the locations of ore deposits, which makes it especially useful in areas that are difficult to access. Currently, two concepts are being explored for this application. The first involves a LIBS system mounted on a rover or robot that scans rocks from afar. The second concept features a LIBS system mounted on a robotic arm, which creates a systematic scanning pattern. Figure 8 below presents LIBS mine-face scanning scheme.

Spectral Industries B.V. (Schieweg 15A, 2627 AN Delft, The Netherlands), has been developing custom LIBS (laser-induced breakdown spectroscopy) systems for various applications, particularly in the mining industry, for over eight years. During this time, Spectral Industries has proposed innovative solutions for designing LIBS systems tailored for conveyor belt scanners, core scanners, and drill chip analyzers. This experience is crucial for the advancement of other novel LIBS applications.

In the initial stage of development, a series of simulations will be conducted to evaluate different sensor designs. The goal is to determine which design performs optimally at a distance while meeting other essential requirements, such as being lightweight. Once the feasibility of the chosen system is confirmed, SPE will create a prototype and test it in a laboratory environment.

To develop a LIBS system suitable for mine-face scanning, several innovative aspects need to be explored. Firstly, the working distance must be significantly increased beyond the current maximum of 0.5 m, which is typical for conventional LIBS systems. This increase will require studying novel optical configurations that enable accurate measurements at long distances. Additionally, the LIBS system must be integrated with a scanning mechanism capable of covering the entire mine face. While several potential methods exist, it is essential to investigate which options are both cost-effective and feasible in the challenging environment of underground mining. Current concepts involve a pan-tilt system for long-distance LIBS systems or a robotic arm system for shorter distance LIBS systems [37,38,39].

Long-distance LIBS sensors have been successfully developed by various research groups in academia and for space applications. However, these systems are generally not designed for practical, real-world applications and often suffer from issues related to cost, size, and durability. The focus of this project will be to design a sensor that meets the stringent requirements of underground mining or at least to conceptualize a scalable solution that can achieve this goal.

## 6. Conclusions

The automation of mining machines, particularly those operating in deep underground mines, is significantly transforming the mining industry. Advances in remote drilling, autonomous navigation, and modular robotic systems have made it possible to revolutionize operations in this field. Autonomous drilling rigs, equipped with real-time monitoring systems, advanced navigation technologies, and communication networks like LoRa WAN, can now function effectively in hard-to-reach and hazardous environments, thus enhancing both efficiency and safety. The PERSEPHONE project represents a major stride in this transformation. It integrates modular fleets of robots outfitted with advanced sensors, modern drill planning systems, and real-time geological analysis technologies to address the unique challenges of working in deep and abandoned underground mines. The collaboration between machines within these decentralized fleet models is innovative and promotes the development of modern drilling technologies. PERSEPHONE optimizes the drilling efficiency by dynamically adapting to various geological conditions in real time. Its continuous monitoring system for borehole deviation, along with rock characterization technologies such as mud analysis and LIBS face scanning, provides unprecedented insights into subsurface conditions. The solutions presented in this project enhance blasting accuracy, reduce operating costs, and improve long-term mine stability.

While the full quantification of sustainability-related benefits will require field validation under real operational conditions, a preliminary assessment framework can already be established to highlight the expected contributions of the PERSEPHONE system to more sustainable underground mining practices. Key areas of anticipated impact include the following:

- Energy efficiency: The ability to adjust the drilling patterns dynamically—based on real-time sensing of rock properties—is expected to reduce unnecessary boreholes and associated energy consumption during drilling operations.
- Reduction in explosive use: By aligning charge placement with localized geomechanical feedback, the system can help optimize fragmentation and limit overbreak, leading to a more efficient use of explosives.
- Minimized human exposure: Through the deployment of autonomous systems for drilling and preconditioning in difficult-to-access or hazardous areas, the need for direct human intervention is expected to be significantly reduced, enhancing overall occupational safety.
- Lower emissions and ventilation demand: The integration of electric-powered autonomous platforms in place of conventional diesel-driven equipment may reduce underground emissions and help optimize ventilation strategies, particularly in deep or poorly ventilated areas.
- Modular reusability and lifecycle extension: The modular architecture of the robotic systems allows for targeted maintenance and upgrades, thereby reducing waste and increasing the longevity of critical hardware components.

Although these criteria remain qualitative at this stage, they provide a structured foundation for future sustainability assessment once operational data becomes available. They also reflect the system’s alignment with broader industry goals toward safer, more efficient, and environmentally responsible mineral exploration.

The PERSEPHONE project not only advances modern autonomous mining solutions but also sets new standards for intelligent, safe, and sustainable deposit exploration in the future. With enhanced automation, onboard sensing, and real-time geological analysis, the system is anticipated to improve the planning and execution of drilling operations, supporting more accurate and targeted blasting strategies. As discussed in the paper, detailed knowledge of the rock face geometry and composition—gained through modular sensors and technologies like LIBS—enables dynamic adjustments to drilling and blasting patterns. While the current conclusions about operational efficiency stem from simulation results and controlled-environment tests, field trials and real-world validation are essential to confirm these performance gains. Therefore, the reported improvements in accuracy and potential cost reductions should be interpreted as expected benefits arising from the system’s technical architecture and integration, rather than as verified results from actual field outcomes.

Similarly, while the system is projected to enhance sustainability through reduced overbreak, minimized human exposure in hazardous areas, and better-targeted exploration, no quantitative metrics for sustainability are currently provided. These impacts will be assessed in future phases of the project, once real-world deployments and operational data become available.

## Figures and Tables

**Table 1:** Failure scenarios and adaptive responses.

Failure Scenario	Detected by	Immediate Action	Adaptive Strategy	Notification/Sync
No Suitable Deployment Site Found	Deployer	Pause deployment	Expand search area or reposition for new perspective	Notify dispatcher and await instructions
Stinger Retrieval Failure	Deployer	Attempt regrasp or reposition	Request supplier to provide another unit	Notify dispatcher; update shared state
Deployment Misalignment	Deployer	Abort deployment	Reposition and attempt precise placement again	Notify stinger and update placement status
Anchoring Failure (Unstable Terrain)	Stinger	Abort anchoring	Request repositioning to alternate site	Notify deployer
Resource Supply Delay or Fault	Stinger/deployer	Retry request for resources	Switch supplier (if available), reschedule operation	Notify dispatcher
Drilling Head Jam or Tool Failure	Stinger	Halt drilling, disengage tool	Attempt self-repair or request tool replacement	Notify deployer and dispatcher
Material Too Hard/Unexpected Composition	Stinger	Pause drilling	Adjust drilling parameters or change bit/tool	Share data with team for future planning
Communication Drop	Any agent	Retry communication	Attempt mesh reconnection or fallback protocol	Alert dispatcher if timeout exceeds threshold
Incomplete Drilling at Site	Stinger	Request repositioning	Deployer relocates robot to alternate site	Confirm new plan with all agents
Repositioning Failure (e.g., Terrain Obstacle)	Deployer	Abort current path	Plan alternative route using updated map	Notify stinger and dispatcher
Mission Phase Validation Timeout	Any agent	Trigger safe state	Retry validation; if failure persists, notify team	Alert dispatcher and wait for manual override

**Table 2:** Comparative overview of elemental analysis methods for mining applications [31,32,34,35,36].

Parameter	LIBS	XRF	Mass Spectrometry (e.g., ICP-MS)
Sample preparation	Minimal or none	Minimal (flattening, drying, grinding)	Complex (acid digestion, filtration)
Acquisition time	Seconds (1–10 s)	Tens of seconds	Minutes (plus extensive sample preparation)
Detection limits	ppm level (matrix-dependent)	sub-ppm to ppm	sub-ppb to ppm
Accuracy	Moderate (±5–20%)	High (±1–5%)	Very high (±0.1–1%)
Portability	High (robot-compatible, compact)	Moderate (handheld units available)	Very low (lab-based only)
Light element detection (e.g., Li)	Yes (effective for low-Z elements)	Limited (detection drops for Z < 11)	Yes
Radiation safety	No special restrictions	Requires control of ionizing radiation	No (chemical hazards apply)
In situ/robotic integration	Excellent (already field-tested)	Limited (requires stability and contact)	Not applicable

## Data Availability

The original contributions presented in this study are included in the article. Further inquiries can be directed to the corresponding author.
